# Gene expression in the brain and kidney of rainbow trout in response to handling stress

**DOI:** 10.1186/1471-2164-6-3

**Published:** 2005-01-06

**Authors:** Aleksei Krasnov, Heikki Koskinen, Petri Pehkonen, Caird E Rexroad, Sergey Afanasyev, Hannu Mölsä

**Affiliations:** 1 Institute of Applied Biotechnology, University of Kuopio, P.O.B. 1627, 70211 Kuopio, Finland; 2 National Center for Cool and Cold Water Aquaculture, USDA-ARS, 11876 Leetown Road, Kearneysville, WV 25430, USA; 3 Sechenov Institute of Evolutionary Physiology and Biochemistry, M.Toreza av. 44, Petersburg, 194223, Russia

## Abstract

**Background:**

Microarray technologies are rapidly becoming available for new species including teleost fishes. We constructed a rainbow trout cDNA microarray targeted at the identification of genes which are differentially expressed in response to environmental stressors. This platform included clones from normalized and subtracted libraries and genes selected through functional annotation. Present study focused on time-course comparisons of stress responses in the brain and kidney and the identification of a set of genes which are diagnostic for stress response.

**Results:**

Fish were stressed with handling and samples were collected 1, 3 and 5 days after the first exposure. Gene expression profiles were analysed in terms of Gene Ontology categories. Stress affected different functional groups of genes in the tissues studied. Mitochondria, extracellular matrix and endopeptidases (especially collagenases) were the major targets in kidney. Stress response in brain was characterized with dramatic temporal alterations. Metal ion binding proteins, glycolytic enzymes and motor proteins were induced transiently, whereas expression of genes involved in stress and immune response, cell proliferation and growth, signal transduction and apoptosis, protein biosynthesis and folding changed in a reciprocal fashion. Despite dramatic difference between tissues and time-points, we were able to identify a group of 48 genes that showed strong correlation of expression profiles (Pearson r > |0.65|) in 35 microarray experiments being regulated by stress. We evaluated performance of the clone sets used for preparation of microarray. Overall, the number of differentially expressed genes was markedly higher in EST than in genes selected through Gene Ontology annotations, however 63% of stress-responsive genes were from this group.

**Conclusions:**

1. Stress responses in fish brain and kidney are different in function and time-course. 2. Identification of stress-regulated genes provides the possibility for measuring stress responses in various conditions and further search for the functionally related genes.

## Background

Until recently multiple gene expression profiling was applied almost exclusively to human and a few model organisms. At present cDNA microarrays are being constructed for new species including teleost fishes [1-6]. Since EST sequencing projects are carried out with a large number of species, continuous development of new platforms can be expected in the future. We designed a salmonid fish cDNA microarray primarily to characterize responses to stress, toxicity and pathogens. This paper focuses on time-course comparisons of stress responses in rainbow trout and the usage of functional annotation to conduct analyses of gene expression data.

Functional annotation of genes, especially Gene Ontology [7] is increasingly being used for analyses and interpretation of microarray results [8-13]. We applied Gene Ontology in several modes to facilitate implementation of our research tasks. Furthermore, experimental results generated guidelines for the development of specialized microarrays. Well designed platforms are expected to ensure identification of differentially expressed genes while containing representative coverage from important functional groups. Custom made microarrays include clones from cDNA libraries and/or selected genes, which have advantages and drawbacks. Indiscriminant spotting of EST may result in under representation of many functional classes. On the other hand selection of genes fully relies on annotations and hypotheses, which can be misleading and limit possibilities for nontrivial findings. We used clones from normalized and subtracted cDNA libraries as well as genes selected by the functional categories of Gene Ontology for inclusion onto a microarray targeted at characterizing transcriptome responses to environmental stressors. Designing a new platform requires balancing a large number of genes versus multiple replications of spots, which enhances statistical analyses of data. The rainbow trout microarray was prepared by spotting of relatively small number of genes (1300) in 6 replicates. We show that multiple replications combined with the dye-swap design of hybridization [14,15] allows for accurate detection of relatively small alterations in expression levels, which is important for the functional interpretation of results.

Stress is closely associated with many diverse issues in fish biology and environmental research (reviewed in [16]). Stress is generally defined as the reaction to external forces and abnormal conditions that tend to disturb an organism's homeostasis. To illustrate the major trends in the studies of stress in fish, we performed a computer-assisted analysis of Medline abstracts covering this area (Table 1). Salmonids have been studied more extensively than any other fish species. Research has focused on various biotic and abiotic factors including toxicity, environmental parameters (oxygen, temperature, salinity, acidosis), diseases, social interactions (crowding, aggressiveness) and farming manipulations. Analysis of Medline abstracts indicated physiological processes, cellular structure and selected proteins that have been the major foci of previous fish stress studies. This provided an outline for interpretation of our results. We analyzed the effects of stress on the transcriptome in the brain and kidney, which are considered important target tissues along with muscle, blood cells, liver and epithelia. We report a profound difference of stress response in these tissues and the identification of a diagnostic set of genes.

**Table 1:** Thematic associations in studies of fish stress. Computer-assisted analysis of 11129 Medline abstracts was performed as described in Methods. Terms that were over-represented in the abstracts (exact Fisher's test, P < 0.05) are ranked by the numbers of occurrence.

**Category**	**Terms (counts/1000 abstracts)**
Species	Salmonids (126.4), carp (68.9), eels (67.0), catfish (37.7), tilapia (38.7)
Stressors	Toxicity (440.6), temperature (178.3), oxygen (91.5), confinement (52.8), salinity (46.2), hypoxia (54.7), diseases (20.8), crowding (23.6), acidosis (17.9), aggressiveness (11.3)
Messengers	Cortisol (208.5), catecholamines (159.4), steroids (92.5)
Tissues	Muscle (197.2), blood cells (152.9), pituitary (119.8), liver (123.6), epithelia (96.2), brain (90.6), kidney (89.6), heart (51.9), skin (42.5)
Cellular structures	Cytosol (42.5), collagen (17.0), cytoskeleton (15.1), microsome (15.1), microtubule (14.2), lysosomes (13.2), peroxisome (4.7)
Oxidative stress	Glutathion (167.9), oxidant (93.4), antioxidant (90.6), peroxide (66.0), radical (55.7), superoxide (40.6), catalase (35.8), redox (18.9)
Other processes	Immunity (91.5), secretion (80.2), metabolism (74), transport (56.6), defense (52.8), necrosis (28.3), apoptosis (18.9), phosphorylation (15.1), proteolysis (7.5)
Metabolites	Ion (987.7), iron (215.1), glucose (141.5), lactate (67.9), lipid (74.5), zinc (51.9), phospholipid (11.3), triglyceride (11.3), lipopolysaccharide (9.4)
Proteins	Enzymes (180.2), heat-shock proteins (84.0), hemoglobin (37.7), metallothionein (37.7), transferase (32.1), phosphatase (26.4), chaperones (21.7), glutathion-S-transferase (17.0), transaminase (17.0), Na/K-ATPase (17.0), aminotransferase (8.5), mitogen-activated kinases (4.7)

## Results

### 1 Design of cDNA microarray

**Figure 1:**
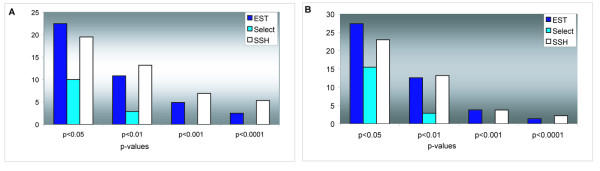
**Performance of the clone sets used for preparation of the microarray. **Figure shows frequencies of genes that were differentially expressed in at least 5 samples at different p-values (Student's t-test). **A: **this study (stress response), **B: **related experiments (exposure to aquatic contaminants [34], response to stress, cortisol and combination of these treatments, challenge with bacterial antigens, M74 disease). SSH – subtracted cDNA libraries, EST – normalized libraries, Select – genes chosen by the Gene Ontology functional categories.

The rainbow trout cDNA microarray was composed of EST and selected genes. The cDNA libraries were prepared from tissues of stressed fish using suppression subtractive hybridization, SSH [17] and a modification of the cap-finder method [18] supplemented with enzymatic normalization [19]. We sequenced 2000 clones and redundancy of the subtracted libraries was markedly greater than that of the normalized (306% and 134% respectively). In addition to EST we selected rainbow trout transcripts from the normalized multi-tissue cDNA library [20] based on their assignment to functional categories of Gene Ontology (stress and defense response, regulation of cell cycle, signal transduction, chaperone activity and apoptosis). The selected genes substantially improved the coverage of many functional classes (Table 2), though the number of differentially expressed genes in this group was markedly inferior to EST (Figure 1). Subtraction cloning enriched genes that showed strong alteration of expression at response to stress (p < 0.01 or lower, Figure 1A), however the SSH clone set did not provide any advantage when microarray was used for the related research tasks (Figure 1B).

**Table 2:** Presentation of the Gene Ontology functional categories in the microarray. Table shows the numbers and frequncies of genes in the clone sets that were used for spotting (SSH – subtracted libraries, EST – normalized libraries).

**Gene Ontology classes**	**N on slide**	**SSH**	**EST**	**Selected**
Response to external stimulus	147	11 (0.07)	48 (0.11)	88 (0.31)
Response to stress	145	7 (0.04)	30 (0.07)	108 (0.38)
Defense response	105	6 (0.04)	34 (0.08)	65 (0.23)
Humoral immune response	42	3 (0.02)	13 (0.03)	26 (0.09)
Apoptosis	79	6 (0.04)	10 (0.02)	63 (0.22)
Cell communication	139	11 (0.07)	45 (0.11)	83 (0.29)
Cell proliferation	82	8 (0.05)	23 (0.05)	51 (0.18)
Cell cycle	62	2 (0.01)	17 (0.04)	43 (0.15)
Signal transduction	114	5 (0.03)	32 (0.07)	77 (0.27)
Receptor activity	49	3 (0.02)	18 (0.04)	28 (0.10)
Intracellular signaling cascade	49	3 (0.02)	15 (0.04)	31 (0.11)
DNA metabolism	47	5 (0.03)	15 (0.04)	27 (0.09)
Transcription	67	9 (0.05)	21 (0.05)	37 (0.13)
Chaperone activity	41	4 (0.02)	12 (0.03)	25 (0.09)

### 2 Stress response in the brain and kidney of rainbow trout

#### 2.1 Differentially expressed genes

**Figure 2:**
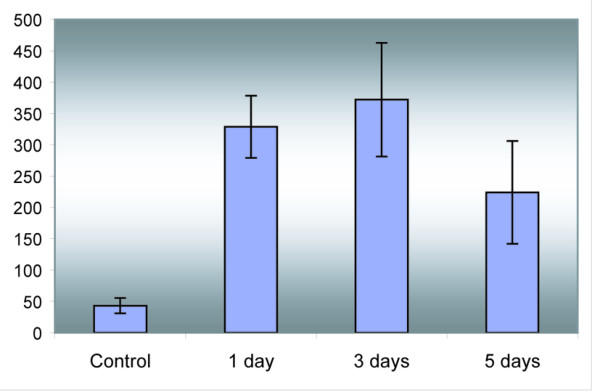
**Plasma cortisol levels. **The data are mean ± SE (n = 4). Difference between the control and stressed fish is significant (Student's t-test, p < 0.05).

Fish were stressed with netting and samples were collected 1, 3 and 5 days after the first exposure. We used plasma cortisol as a stress marker [21]. The hormone levels increased 7.6-fold after 1 day and did not change significantly to the end of experiment (Figure 2).

**Figure 3:**
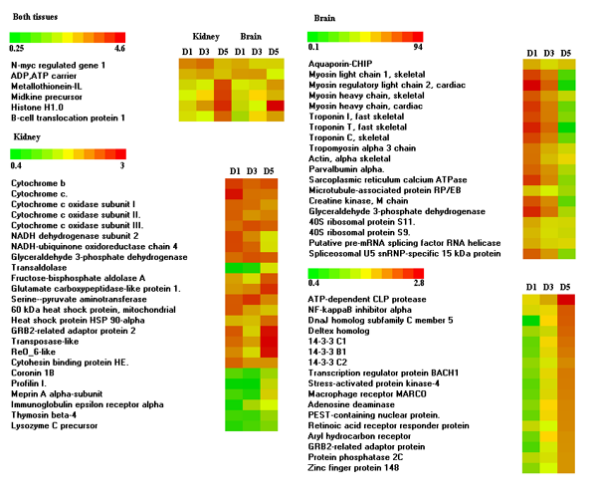
**Examples of differentially expressed genes**. Pooled RNA from 4 fish was hybridized in dye-swap experiments to two microarrays on which each gene was printed 6 times (total of 12 replicates). Differential expression was analysed with Student's t-test (P < 0.01); the expression ratio is coded with color scale.

Microarray results were submitted to GEO (GSM22355). Two genes were up-regulated in both tissues at all time-points (Figure 3). One is a putative homolog to the mammalian N-myc regulated genes, which are induced with steroid hormones in the brain [22] and kidney [23]. Mitochondrial ADP, ATP carrier can be implicated to both normal functions and cell death [24]. Metallothionein-IL, a classical stress marker was induced to the end of experiment and a similar profile was seen in midkine precursor (growth factor), histone H1.0 and B-cell translocation protein 1. In kidney we observed consistent up-regulation of genes related to energy metabolism, such as mitochondrial proteins (cytochromes b and c, cytochrome oxidases), enzymes (glyceraldehyde 3-phosphate dehydrogenase, fructose-bisphosphate aldolase, serine-pyruvate aminotransferase) and similar profiles were seen in two heat shock proteins and two signal transducers (cytohesin binding protein and GRB2-adaptor). The repressed genes were related to actin binding (coronin and profilin) and immune response (meprin, immunoglobulin epsilon receptor, thymosin and lysozyme).

Rapid alteration of gene expression was a remarkable feature of stress response in the brain. Only one gene, aquaporin, was up-regulated for the duration of the experiment. Water channel aquaporin plays a key role in water homeostasis being implicated in various physiological processes and pathological conditions [25]. A panel of genes which showed markedly increased expression after 1 day was also suppressed after 5 days. Surprisingly, this group included mainly genes that are predominantly expressed in skeletal or cardiac muscle (myosin light chain 1 and 2, skeletal and cardiac isoforms, myosin heavy chain, troponin I, T and C) or are involved in regulation of muscle contraction (parvalbumin alpha and sarcoplasmic reticulum calcium ATPase). An opposite tendency was shown by a large group of genes however the magnitude of expression changes was smaller. We analysed 5 differentially expressed genes with qPCR and the results were in close concordance with the microarray data (not shown).

#### 2.2 Functional classes

The search for enriched Gene Ontology functional categories in the lists of differentially expressed genes found almost no overlap between the tissues (Table 3). In the brain stress affected binding and transport of metal ions, especially calcium and manganese, chaperones and heat shock proteins, cytoskeleton and microtubules and a number of signaling pathways; whereas, mitochondrion, extracellular structures and peptidases appeared the primary targets in the kidney.

**Table 3:** Enrichment of Gene Ontology categories in the lists of differentially expressed genes. Analysis with exact Fisher's test, (p < 0.05) was made using the composition of microarray as a reference. The numbers of differentially expressed genes and genes on the microarray are in parentheses.

**Brain**	**Kidney**
Intracellular signaling cascade (19/47)	Mitochondrion (19/71)
RAS protein signal transduction (6/9)	Electron transporters (13/43)
GTPase mediated signal transduction (11/16)	Extracellular (19/70)
Chaperones (16/40)	Endopeptidases (8/22)
Heat shock proteins (8/16)	Metallopeptidases (7/12)
Metal ion binding (31/80)	Zinc ion binding (8/24)
Carriers (15/37)	
Potential-driven transporters (7/9)	
Calcium ion binding (20/41)	
Magnesium ion binding (8/14)	
Cytoskeleton (27/76)	
Myofibril (16/16)	
Microtubule-based process (6/6)	

**Figure 4:**
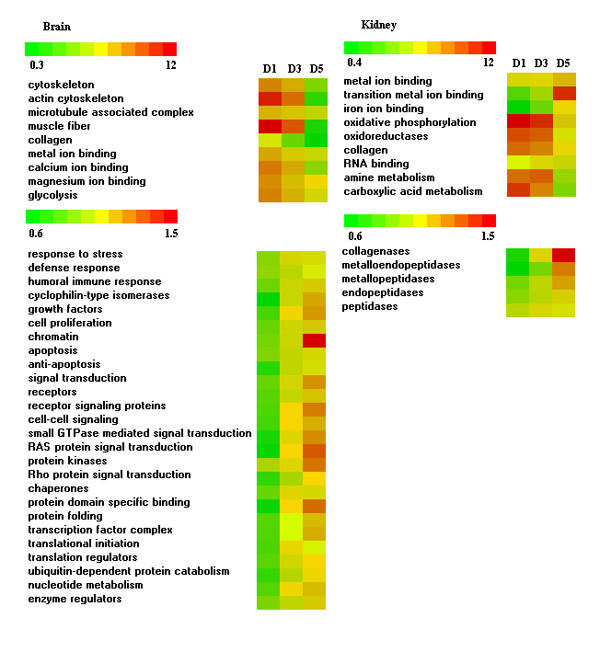
**Time-course of stress response in the brain and kidney**. Differentially expressed genes were grouped by the Gene Ontology categories and mean log (expression ratios) were analysed with Student's t-test. Panel presents examples of categories that showed significant difference between the time points (p < 0.05). The values are coded with color scale.

Comparison of the differentially expressed genes by the Gene Ontology categories suggested coordinated regulation of various cellular functions in the brain. Early stress response was marked with transient induction of the cytoskeleton proteins and similar profiles were observed in the metal binding proteins and enzymes of carbohydrate metabolism (Figure 4). An opposite expression pattern was shown by a large group of genes involved in stress and immune response, regulation of growth and cell cycle, apoptosis, signal transduction and cell to cell signaling. This was in parallel with enhancement of transcription and translation, ubiquitin-dependent protein catabolism and protein folding. In the kidney the temporal alterations were much weaker. Expression of metal binding proteins increased slowly in parallel with peptidases. Strong induction of collagenases coincided with decrease of collagen expression. At the same time a number of metabolic functions were suppressed (oxidative phosphorylation and oxidoreductase activity, amine metabolism and RNA binding).

### 3 Stress-responsive genes

**Figure 5:**
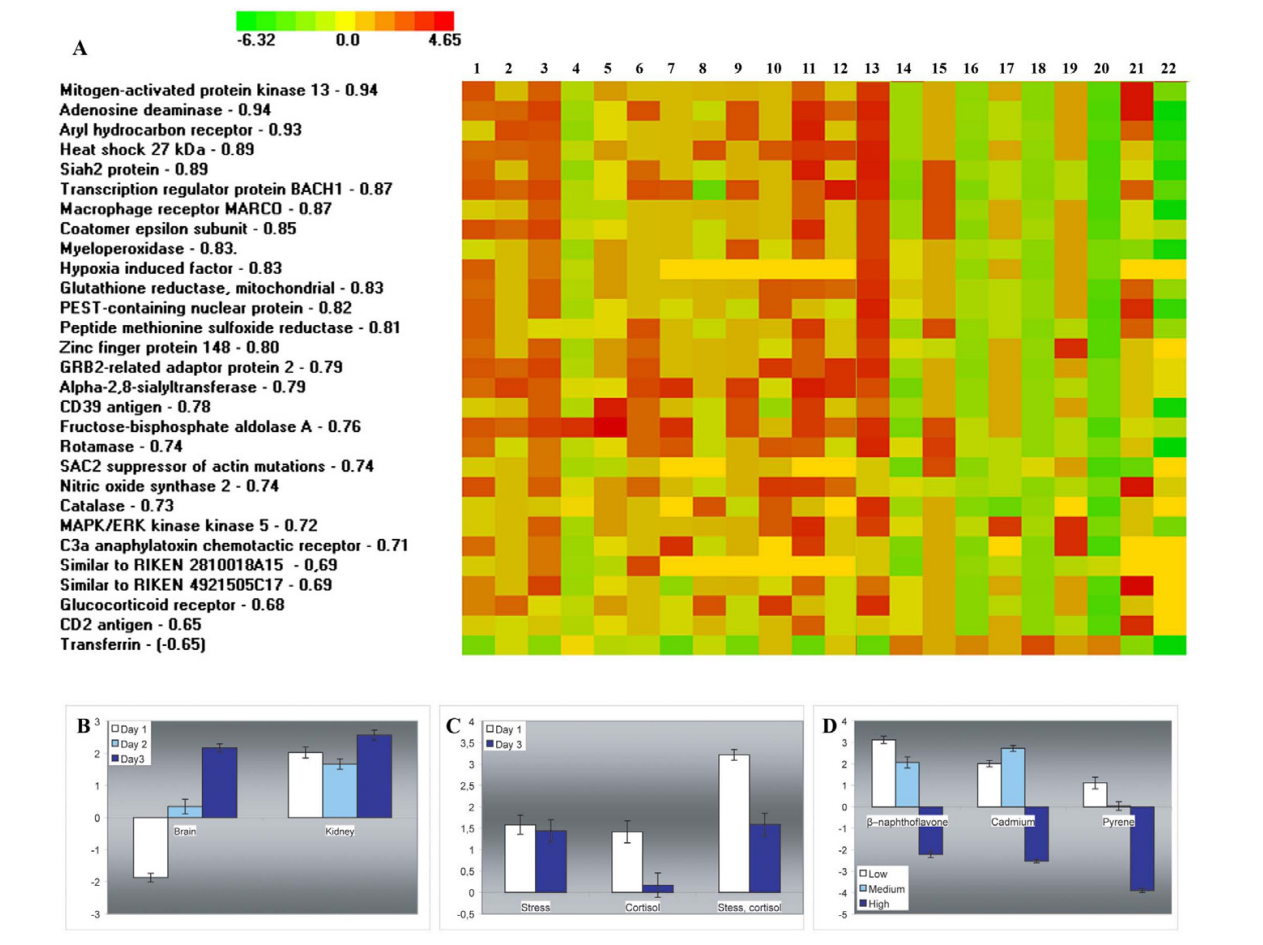
**Expression of stress-responsive genes**. **A**: Experiments. **1–6**: response to handling stress, this study. Kidney, 1 day (1), 3 days (2) and 5 days (3); brain, 1 day (4), 3 days (5) and 5 days (6). **7–12**: response to handling stress and exogenous cortisol in kidney. Cortisol, 1 day (7) and 3 days (8); stress, 1 day (9) and 3 days (10), combination of stress and injection of cortisol, 1 day (11) and 3 days (12). **13–20**: exposure of yolk sac fry to model contaminants [34]. β-naphthoflavone, low (13) and high (14) dose; cadmium, low (15) and high (16) dose; carbon tetrachloride, low (17) and high (18) dose; pyrene, low (19) and high (20) dose. **21–22: **response of yolk sac fry to transportation stress, rainbow trout (21) and Atlantic salmon (22). Ranks are coded with color scale; correlation coefficients (Pearson r) with the mean expression profile are indicated. **B-C: **the mean ranks ± SE of the stress-responsive genes in 3 experiments. **A**: this study; **B **– response to handling stress and injection of cortisol in kidney; **C **– exposure of yolk sac fry to β-naphthoflavone, cadmium and pyrene at low, medium and high doses.

Microarray design included genes from functional categories which were expected to be affected by stress (Table 2). Overall observations of differences in gene expression from this group in response to handling stress were minimal; however, this could be accounted for by its heterogeneity. Therefore we searched for the subgroups of genes with correlated expression profiles within the functional classes using results of 35 microarray experiments conducted by our laboratory. Both factorial and cluster analyses revealed 9 defense response genes that showed tightly coordinated expression being induced with stress. We continued search using the consensus profile of this subgroup and found 47 positively and 1 negatively correlated genes (Pearson r > |0.65|). Of these 29 were identified by the protein products (Figure 5A), 19 being from the set of selected clones. Expression of the stress-responsive genes changed significantly in several experiments including this study (Figure 5B). They were up-regulated in kidney with stress and injection of cortisol, combination of these treatments showed an additive effect (Figure 5C). These genes also responded to the model water contaminants, being induced with low and medium and repressed with high doses (Figure 5D).

## Discussion

### I Stress response in rainbow trout

Our study aimed at comparison of time-course of stress response in rainbow trout brain and kidney and finding of a diagnostic set of genes. These tasks were implemented with an aid of Gene Ontology annotation, which was used in several modes. The most straightforward and commonly used approach is counting of Gene Ontology classes in the lists of differentially expressed genes. Statistical inference of enrichment and depletion is made with Z-score of hypergeometric distribution, exact Fisher's test or its modifications. Such analyses helped us to interpret differences of stress responses in the brain and kidney (Table 3). In the brain handling stress mainly affected expression of transcripts for structural proteins (especially cytoskeleton), signal transduction, and binding of metal ions, whereas mitochondria, extracellular structures and peptidases appeared the key targets in the kidney. Computer-assisted analysis of Medline abstracts suggested that most of these themes have not been addressed in the studies of fish stress (Table 1).

Searches of the enriched Gene Ontology categories associated with differentially expressed transcripts is useful for rapid screens of microarray data; however, it presumes coordinated expression of functionally related genes. This assumption is not valid for many classes, especially large and heterogenous groups, such as stress and defense response. Because the gene composition of microarray is used as a reference, uneven presentation of functional categories can distort the results. Finally, this analysis does not take into account direction and magnitude of differential expression. To overcome these problems, enrichment of Gene Ontology classes is analysed in groups of genes with similar expression profiles revealed with cluster or factorial analyses. In this study we preferred straight comparison of Gene Ontology classes by the mean log expression ratios which helped in interpretation of the time-course of stress response.

In the kidney temporal alterations were relatively weak though significant. Expression of peptidases (especially collagenases) increased steadily, which implied possible degradation of tissue with prolonged stress. We could expect abrupt fluctuations in the rainbow trout brain, since transient induction and up-regulation of gene expression was observed in response to cold in the brain of channel catfish [4]. In our study most differentially expressed genes fell into two groups with distinct temporal profiles which showed remarkable coherence of the functional classes. Early phase was associated with dramatic up-regulation of structural and metal binding proteins, which were repressed in later phases. Expression of genes involved in stress and defense response, apoptosis and signal transduction, cell cycle and growth changed in a reciprocal fashion. Activation of metal binding proteins could be accounted for the role of ions (particularly calcium) in multiple pathways of gene expression regulation in the brain [26]. Motor proteins of cytoskeleton play key roles in the transport of vesicles and the establishment and rearrangement of neuronal networks [27-29] which also could be implicated to the stress response in fish. However, in mammals these functions are associated with non-muscle isoforms and therefore differential expression of the sarcomeric proteins was unexpected. Additional experiments confirmed induction of these proteins at early phase of stress response. Previously we observed high activity of skeletal α-actin and myosin light chain 2 promoters in the neural tissues of rainbow trout embryos [30]. Sequencing of salmonid fish cDNA libraries provided evidence for the brain expression of sarcomeric proteins, but their role remains fully unknown. At present there is sparse evidence for differential expression of structural muscle proteins in the mammalian brain. For example regulation of troponin I with dextromethorphan (antagonist of excitatory amino acid receptors) was reported in the rat hyppocampus and cortex [31].

Grouping of individual differentially expressed genes by the functional classes reduced noise and enhanced cluster and factorial analyses. This helped to identify stress-responsive genes that showed correlated expression in 35 microarray experiments (22 experiments are shown in Figure 5A). Association with stress is well established for most of these proteins and some are used as stress markers. The list of enriched Gene Ontology categories (stress, defense and humoral immune response, signal transduction and response to oxidative stress, p < 0.05) suggested biological relevance of this group. Computer analysis of Medline abstracts (Table 1) showed that immunity and metabolism of reactive oxidative species are prioritized in studies of fish stress and these functional categories were enriched in the list of stress-responsive genes. Thus Gene Ontology provided a useful starting point for search of functionally related genes and results of these analyses can be used further for the revision of annotations.

### 2 Construction of microarrays

Results of our experiments helped to evaluate the strategy used in construction of the rainbow trout microarray. Researchers developing microarrays for new species are commonly choosing between specially selected genes and clones from normalized and subtracted cDNA libraries. We used SSH, which is at present probably the most popular method of subtraction. Though proven efficient in many studies, this method has a number of drawbacks. Subtraction requires re-association of tester and formation of double-stranded DNA, hence many rare transcripts are not cloned and variations in concentrations of cDNA and hybridization conditions may have strong impacts on library composition. High redundancy is a common feature of the SSH libraries. Apart from these problems, rapid alterations of gene expression observed in this study and many other microarray experiments make the advantages of subtractive cloning ambiguous. Subtraction achieves enrichment of the transcripts, which are over or under represented in the test sample. In many cases one sample will not provide coverage of differentially expressed genes for the whole series, whereas pooling of samples may reduce fluctuations. Furthermore, we observed relatively high ratio of differentially expressed genes among the clones from the unsubtracted cDNA libraries, which are easier for construction and much less redundant. The advantage of subtractive cloning becomes negligible when microarrays are used for different, though related research tasks (Fig. 1B).

At present selection of genes for microarrays is facilitated with advances of functional annotation. This helped us to improve presentation of many functional categories (Table 1) and enhanced interpretation of results. Most of the selected genes did not show differential expression in our studies, however 63% of stress-responsive genes were from this group. In our view, this finding is a strong argument for utilizing Gene Ontology in the development of specialized platforms.

Given the limited number of spots on slides, microarray design requires a careful balance between the number of genes and replication of spots. Apparent advantages of genome-wide platforms are compromised with the problems associated with identifying significantly differentially expressed genes. We preferred combination of multiple spotting and dye-swap normalization, which ensured robust normalization and accurate detection of differential expression at low ratios. Coordinated expression of functionally related genes suggested biological relevance of relatively small alterations in the transcription levels. Selection of differentially expressed genes by the cutoff values would result in loss of valuable information in our experiments. For instance, most of the stress-responsive genes showed small or moderate expression changes, the identification of this group would not be likely without multiple replications.

## Conclusions

1. Combination of EST and selected genes appears a reasonable way for construction of cDNA microarrays. Multiple replications of spots and dye swap design of hybridization ensure robust normalization and high power of statistical analyses. Finding of differential expression at small ratios is essential for the functional interpretation of microarray data.

2. Stress response in fish brain and kidney is different both by the target functions and time-course. In brain slow progression of adaptive response was preceded with dramatic transient induction of motor and metal ion binding proteins. Prolonged stress was likely to result in slow degradation of extracellular matrix in kidney.

3. Finding of stress-responsive genes provides possibility for measurement of stress in various conditions and search for the functionally related genes.

## Methods

### 1. Computer-assisted analysis of Medline abstracts

Search of Medline was made with queries: "fishes AND stress" (1060 abstracts) and "fishes NOT stress" (10069 abstracts). Abstracts were split into separate words and a list of non-redundant terms was composed. The numbers of abstracts including each term were estimated. The terms were ranked by the Z-scores of hypergeometric distribution and enrichment was analysed with exact Fisher's test (p < 0.05).

### 2. Experiments with fish, exposure and sampling

One year old rainbow trout were stressed with netting for 2 min, this treatment was repeated once a day for a duration of 5 days. Fish were killed with over-dose of anaesthetic (MS-222) and blood was taken from the caudal vein. The kidneys and brains were snap-frozen in liquid nitrogen. Plasma cortisol was determined with RIA using Orion Spectra Cortisol kit.

### 3. Preparation of microarrays

RNA was extracted with Trizol reagent (Invitrogen) and mRNA was purified with Dynabeads kit (Dynal). SSH cloning was performed as described [17]. For preparation of normalized libraries, synthesis of cDNA with PowerScript reverse transcriptase (Clontech) was primed with oligonucleotides including *EcoRI *and *NotI *sites: 5'-ACGAGGCGAATTCACAGAGAGGAG(T)VN-3', 5'-GAGAGAGAGTGGTGCGGCCGCGGTGTATGGGG-3'). Double-stranded cDNA was generated using Advantage DNA polymerase mix (Clontech) and PCR primers: 5'-ACGAGGCGAATTCACAGAGAGGAG-3' and 5'-GAGAGTGGTGCGGCCGCGGTGTA-3'. The PCR products were purified with QIAquick kit (Qiagen), precipitated with ethanol and dissolved to 1 μg/μl in hybridization buffer (1 M NaCl, 50 mM HEPES (pH 8.3), 1 mM EDTA). DNA was denaturated for 5 min at 94°C. Following re-association at 72°C for 16 hours, DNA was ethanol precipitated and digested with 150 U of exonuclease III (MBI Fermentas) for 15 min at 37°C. This treatment eliminates re-associated double-stranded DNA [19]. Single-stranded DNA was PCR amplified, size separated with agarose gel electrophoresis and cloned into pGEM^®^-11Zf (+) (Promega). Normalized and subtracted cDNA libraries were prepared from the stressed fish (whole fry, brain, kidney and spleen of 1-year old fish). A number of clones were from the rainbow trout and Baltic salmon cDNA libraries constructed in University of Turku. The sequences were analysed with stand-alone blastn and blastx [32] Microarray incldued 315 genes selected by the Gene Ontology functional categories. Of these, 282 clones were from the normalized multi-tissue library [20] and the rest were produced with RT PCR. The cDNA inserts were amplified with PCR using universal primers and purified with Millipore Montage PCR96 Cleanup Kit. DNA was spotted onto poly-(L) lysine-coated slides and each clone was printed in 6 replicates.

### 4. Microarray analyses

Total RNA was extracted with Trizol reagent (Invitrogen) and 4 individuals were pooled in each sample. Stressed fish was compared with time-matched control. Labeling with Cy3- and Cy5-dCTP (Amersham Pharmacia) was made using SuperScript III (Invitrogen) and oligo(dT) primer; cDNA was purified with Microcon YM30 (Millipore). We used a dye swap experimental design [14,15] and each sample was hybridized to two microarrays. For the first slide, test and control cDNA were labeled with Cy5 and Cy3 respectively, and for the second array dye assignments were reversed. The slides were pretreated with 1% BSA, fraction V, 5 x SSC, 0.1% SDS (30 min at 50°C) and washed with 2 x SSC (3 min) and 0.2 x SSC (3 min) and hybridized overnight in cocktail containing 1.3 x Denhardt's, 3 x SSC 0.3% SDS, 0.67 μg/μl polyadenylate and 1.4 μg/μl yeast tRNA. All chemicals were from Sigma-Aldrich. Scanning was performed with ScanArray 5000 and images were processed with QuantArray (GSI Luminomics). The measurements in spots were filtered by criteria *I/B ≥ 3 *and (*I*-*B*)/(*S*_*I*_+*S*_*B*_) ≥ *0.6*, where *I *and *B *are the mean signal and background intensities and *S*_*I*_, *S*_*B *_are the standard deviations. After subtraction of mean background, lowess normalization [33] was performed. Differential expression was analysed with Student's t-test (p < 0.01) and the genes were ranked by the log(p-level).

### 5. Quantitative RT PCR

Primers (Table 4) were designed to amplify 194–305 b fragments. RNA was processed with Rnase-free Dnase (Promega). Synthesis of cDNA with Superscript III reverse transcriptase (Invitrogen) was primed with oligo(dT). Analyses were carried out using Dynamo SYBR Green kit (Finnzymes) and ABI Prism 7700 (Amersham-Pharmacia).

**Table 4:** Primers used for qPCR.

**Gene**	**Sequence**
GRB2-related adaptor protein 2	Forward 5'-GCCAGAGCACCCCAGGAGAT-3' Reverse 5'-GGCTGAGAGGATGGGGCTGA-3'
Collagenase type IV	Forward 5'-AACATCAGAAACGCCCTCAT-3' Reverse 5'-TGGTGGTAGTGGTAGTGGAC-3'
Troponin T	Forward 5'-TGGGAAGAAGGAAACTGAGA-3' Reverse 5'-CTCTTACGCAGGGTTGTGAC-3'
40S ribosomal protein S12	Forward 5'-AGACCGCACTCATCCACGAC-3' Reverse 5'-CCACTTTACGGGGTTTTCCT-3'
EST1	Forward 5'-CGGAGAAGGAGAACCCACAG-3' Reverse 5'-CCCTCAAACAAGCAAAGTG-3'
EST2	Forward 5'-GCAAATGACAGCCCTCTTAG-3' Reverse 5'-AGCAGGTTTTCATCAAGGA-3'

## Author's contributions

AK designed microarray, carried out experiments with fish and data analyses and drafted the manuscript. HK conducted the microarray analyses. PP developed software for annotation of genes and analyses of Medline abstracts. CR constructed the multi-tissue cDNA library and provided the selected genes. SA developed software for management of microarray data and performed the statistical analyses. HM coordinated research. All authors read and approved the final manuscript.
